# Innovative organotypic in vitro models for safety assessment: aligning with regulatory requirements and understanding models of the heart, skin, and liver as paradigms

**DOI:** 10.1007/s00204-018-2152-9

**Published:** 2018-01-23

**Authors:** Chris S. Pridgeon, Constanze Schlott, Min Wei Wong, Minne B. Heringa, Tobias Heckel, Joe Leedale, Laurence Launay, Vitalina Gryshkova, Stefan Przyborski, Rachel N. Bearon, Emma L. Wilkinson, Tahera Ansari, John Greenman, Delilah F. G. Hendriks, Sue Gibbs, James Sidaway, Rowena L. Sison-Young, Paul Walker, Mike J. Cross, B. Kevin Park, Chris E. P. Goldring

**Affiliations:** 1 Department of Molecular and Clinical Pharmacology, University of Liverpool, Liverpool, L69 3GE UK; 2 National Institute for Public Health and the Environment (RIVM), Bilthoven, The Netherlands; 3 Dr. Johannes Heidenhain GmbH, Dr.-Johannes-Heidenhain-Straße 5, 83301 Traunreut, Germany; 4 Department of Mathematical Sciences, University of Liverpool, Liverpool, L69 7ZL UK; 5 Technologie Servier, Orléans, France; 6 Investigative Toxicology, Department of Non-Clinical Development, UCB Biopharma SPRL, 1420 Braine L’Alleud, Belgium; 7 Department of Biosciences, Durham University, Durham, DH1 3LE UK; 8 Northwick Park Institute for Medical Research, Northwick Park and St Mark’s Hospital, Middlesex, HA1 3UJ UK; 9 School of Life Sciences, University of Hull, Hull, HU6 7RX UK; 10 Section of Pharmacogenetics, Department of Physiology and Pharmacology, Karolinska Institutet, Stockholm, Sweden; 11 Department of Dermatology, VU University Medical Center, Amsterdam, The Netherlands; 12 Department of Oral Cell Biology, Academic Center for Dentistry Amsterdam, University of Amsterdam and VU University, Amsterdam, The Netherlands; 13 Phenotox Ltd, Cheshire, SK10 5QT UK; 14 Cyprotex Discovery Ltd, Cheshire, SK10 4TG UK

**Keywords:** 3D in vitro models, Heart, Skin, Liver

## Abstract

The development of improved, innovative models for the detection of toxicity of drugs, chemicals, or chemicals in cosmetics is crucial to efficiently bring new products safely to market in a cost-effective and timely manner. In addition, improvement in models to detect toxicity may reduce the incidence of unexpected post-marketing toxicity and reduce or eliminate the need for animal testing. The safety of novel products of the pharmaceutical, chemical, or cosmetics industry must be assured; therefore, toxicological properties need to be assessed. Accepted methods for gathering the information required by law for approval of substances are often animal methods. To reduce, refine, and replace animal testing, innovative organotypic in vitro models have emerged. Such models appear at different levels of complexity ranging from simpler, self-organized three-dimensional (3D) cell cultures up to more advanced scaffold-based co-cultures consisting of multiple cell types. This review provides an overview of recent developments in the field of toxicity testing with in vitro models for three major organ types: heart, skin, and liver. This review also examines regulatory aspects of such models in Europe and the UK, and summarizes best practices to facilitate the acceptance and appropriate use of advanced in vitro models.

## Introduction

When developing novel drugs, chemicals, or personal care products, industry must evaluate the risks to human health arising from their use. Therefore, knowledge of the properties of these substances, results of safety tests, risk assessments, and appropriate measures to adequately control the risks must be provided to regulatory authorities (Eichler et al. 2008; Pignatti et al. 2011; Senderowicz 2010; Silbergeld et al. 2015). This information is mandatory for registration and marketing approval as well as for approval of clinical trials for drugs or personal care products.

**Fig. 1:**
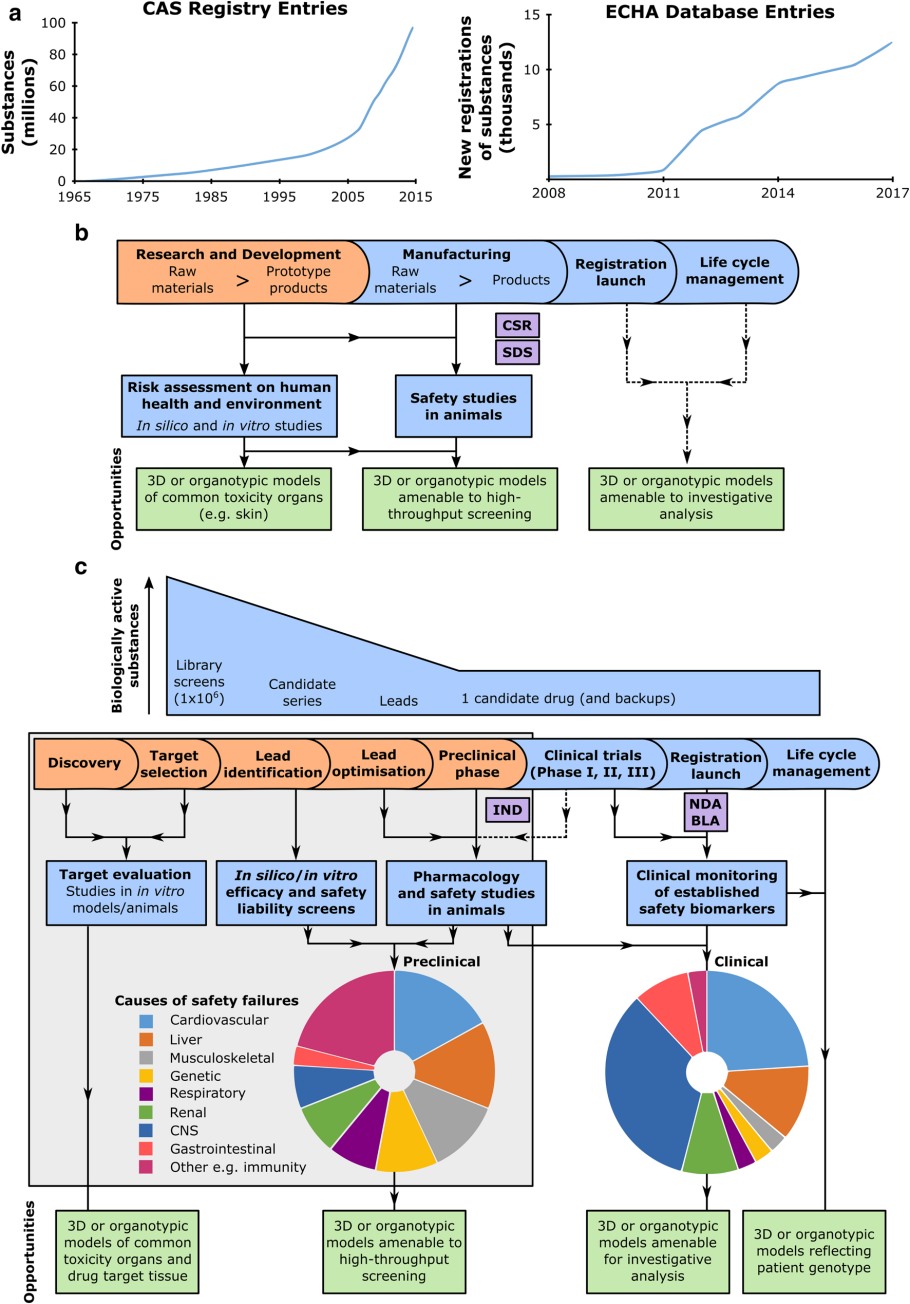
Safety assessments during the development of chemicals or drugs and opportunities for the application of innovative in vitro models. **a** Number of newly synthesized organic and inorganic chemical substances recorded in the CAS Registry (Kemsley 2015) and number of substances registered at the European Chemicals Agency (ECHA). **b** Schematics of the development process for chemical substances including safety assessments. Violet squares show interactions with regulatory authorities with respect to chemical safety reports (CSR) and the provision of safety data sheets (SDS). Opportunities for the application of 3D or organotypic in vitro models are indicated. **c** Schematics of the drug development pipeline from the identification of safety liabilities during discovery, screening, and early development to risk/benefit assessments during clinical trials and product life cycle management. Violet squares show important interactions with regulatory authorities, e.g., Investigational New Drug (IND) applications and New Drug or Biologic License Applications (NDA/BLA). Indicated are the major organ systems involved in pre-clinical and clinical safety failures (Cook et al. 2014) as well as opportunities for the application of 3D or organotypic in vitro models. (Color figure online)

Traditionally, such risk assessments are based on safety tests performed in animals and assume that animals will respond to these tests in a similar manner to humans. Although animals represent systemic organisms with obvious similarities in physiology and function to humans, there are also several limitations. Animal testing is labour-intensive, time-consuming, expensive, ethically challenging and not suited to address the high number of substances produced by the chemical industry or during drug screening in the pharmaceutical industry (Fig. 1) (Hartung 2010; Kessel and Frank 2007). Furthermore, known species variation makes reliance on tests in a single species insufficient for approval of clinical trials in humans (Zbinden 1993). This is why regulatory authorities for pharmaceuticals require in vivo testing in two species, commonly a rodent such as rat or mouse and a non-rodent such as mini-pig, dog, or cynomolgus monkey (Bode et al. 2010; Greaves et al. 2004).

The burden placed upon animal testing has been a contentious subject for many decades, with organized opposition since the nineteenth century (Finn and Stark 2015). Similar ideals are proposed in the EU, UK, US, and in particular by the British National Centre for the Replacement, Refinement and Reduction of Animals in Research (NC3Rs) who follow the principles of humane experimental technique conceptualized by Russell and Burch (Russell et al. 1959). Validated in vitro tests for phototoxicity, cytotoxicity, genotoxicity, and cardiac arrhythmias are already well established to test for cell and DNA damage as well as for cardiac ion channel inhibition. These tests are based on simple cell culture systems employing mammalian cells for photo- and cytotoxicity screens, bacteria, and mammalian cells for the Ames mutagenicity test and in vitro micronucleus test (MNT), or cell lines expressing the human potassium channel hERG in cardiac safety assays (Bridgland-Taylor et al. 2006; Colatsky et al. 2016; Kirkland et al. 2014; Moeller et al. 2012; Shukla et al. 2010; Spielmann et al. 2008).

Testing with in vitro assays and animal tests is relatively effective in the detection of acute and severe toxicities and to some degree in testing for chronic toxicities. However, these tests still exhibit limitations, since ~ 1/3 of candidate drugs fail during clinical trials due to unpredicted toxicity (Arrowsmith and Miller 2013; Cook et al. 2014). Many of these safety failures can be attributed to cardiovascular and liver toxicities (Fig. 1c) (Cook et al. 2014; Laverty et al. 2011). An exemplar case of unexpected toxicity is the clinical trial of the antiviral agent Fialuridine in 1992, in which unforeseen toxicity led to the death of a third of the patient cohort due to liver failure associated with lactic acidosis. Two of the remaining patients required a liver transplant. This toxicity is very pertinent as it was not uncommon in humans, and demonstrates the predictive limitations of pre-clinical studies (McKenzie et al. 1995). More recent examples include the TGN1412 trial, where despite the use of animal studies for the novel immunomodulatory antibody CD28 ‘superagonist’ and use of a dose 500 times lower than found to be safe in animals, all six of the patients suffered from cytokine storm and were hospitalised (Suntharalingam et al. 2006). In addition, animal studies were not predictive of the toxicity observed in first-in-man trials for the fatty acid amide hydrolase inhibitor BIA 10-2474, where five patients suffered neurological injuries and a sixth died (Moore 2016). These failures may be due to a lack in predictivity due to the phylogenetic distance between laboratory animals and humans, as well as the discrepancy between simplistic in vitro tests and the in vivo situation. Animals are not fully predictive of human toxicity and in vitro tests on the traditional two-dimensional (2D) monolayers of cells are neither physiological nor systemic.

Three-dimensional (3D) cell culture and organotypic in vitro models are another approach to bridge the gap between traditional 2D cell culture models and the in vivo situation. Three-dimensional culture produces cells with more physiologically relevant attributes, such as cell polarization, cell–cell or cell–microenvironment interactions, lumen formation, reduced proliferation, increased differentiation, and numerous changes in RNA and protein expression (Edmondson et al. 2014; Kenny et al. 2007; Rimann and Graf-Hausner 2012; Yamada and Cukierman 2007). Therefore, these models hold promise to better represent the histological and physiological complexity of real tissue to study toxicological effects during product development and life cycle management (Fig. 1). The need for innovative models has become particularly urgent for the cosmetics industry following a complete ban on cosmetics developed through animal testing in the European Union since 2013 (EU Regulation no. 1223/2009). Consequently, human in vitro skin equivalents are probably the most developed and understood in vitro engineered 3D model for compound testing (Mathes et al. 2014).

In this review, we discuss innovative in vitro models currently being used or recently developed as well as the regulatory perspective for toxicological safety assessments in the pharmaceutical, chemical, or cosmetics industry, to draw up recommendations for the way forward.

## Regulation

The limitations of animal testing and 2D in vitro systems demonstrate a clear need for better models that can be accepted by regulatory agencies. For example, the EU ban on animal testing for cosmetics and the ambition of the Dutch government to phase out the use of laboratory animals for regulatory safety testing by 2025 (Netherlands National Committee for the protection of animals used for scientific purposes 2016) necessitate regulatory acceptance of alternative methods. Alternative cell culture methods have already emerged in the industry for compound screening prior to regulatory testing. A first aspect to consider in regulatory acceptance is whether legal frameworks allow alternative methods.

Toxicological data requirements for the evaluation and admission of chemical substances on the European market are given in 11 European regulatory frameworks. Analyses of these frameworks has revealed that although most frameworks name certain animal tests as standard for providing certain toxicological information (e.g., for repeated dose toxicity), all but one clearly provide for using alternative methods to obtain this information (Heringa et al. 2014; Vonk et al. 2015). The exception is the framework for veterinary medicinal products, where the legal status of such a possibility is unclear, as this is only provided in a non-binding guideline. In summary, there are no legal barriers to omit animal safety tests during the safety assessment of a novel chemical entity.

The possibility to acquire regulatory acceptance for clinical trial applications without animal safety testing was illustrated in 2010 by the biotechnology company Immunocore Ltd, who received approval for clinical trials in melanoma patients with an immunostimulating biological without animal testing (Megit 2011). In their dialogue with the British Medicines and Healthcare products Regulatory Agency (MHRA) and US Food and Drug Administration (FDA), immunocore brought the arguments forward that their biological can only bind and show activity with human cells and that a relevant animal model is not available for safety evaluation, and that in this case, animal tests have no value. Therefore, it was concluded that extensive testing with human cells and human tissues was sufficient and that toxicity studies in non-relevant species may be misleading and are discouraged, which is in agreement with the International Regulatory Guideline ICH Topic S6: Pre-clinical Safety Evaluation of Biotechnology-Derived Pharmaceuticals (CPMP/ICH/302/95). The MHRA stresses that deviations from the standard safety package are possible when well-justified, which is evaluated on a ‘case-by-case’ basis. The agency recommends that companies consult for advice on the appropriateness of their development programmes before conducting unnecessary and potentially misleading studies. The MHRA, therefore, offers a ‘safe harbour’ approach to researchers presenting new models who may otherwise be concerned about punitive action from regulators. Indeed, where new models are being developed, regulators should be viewed as an ally rather than a hindrance to progress.

This, and the legal situation, is encouraging for the application of alternative models, but raises the question as to why animal testing for safety evaluations have not yet been replaced. The main reason is the lack of scientifically acceptable and physiologically relevant alternatives for animal safety tests. Before approval, a thorough validation is necessary, ideally followed by acceptance within the OECD to achieve mutual recognition of data worldwide.

The validation of alternative tests is the process by which the reliability and relevance of a test are established for a particular purpose (Balls et al. 1990). Over the last several decades, the European centre for validation of alternative methods (ECVAM), in co-operation with international experts, has set up guidelines for validation. Seven modules are proposed for the validity assessment of a test: test definition (scientific purpose and mechanistic basis), intra-laboratory variability, transferability, inter-laboratory variability, predictive capacity, applicability domain, and performance standard (Hartung et al. 2004).

**Fig. 2:**
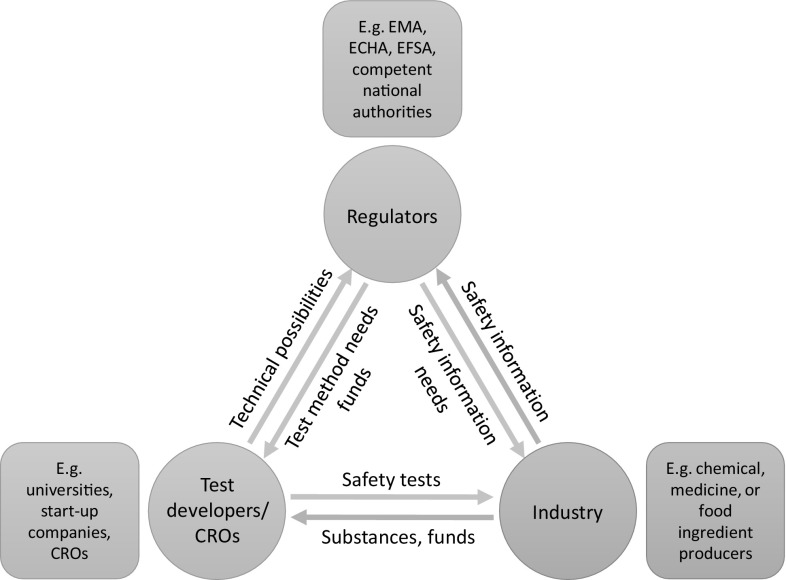
Scheme of the three main actors in the development of safety test methods for chemical substances and their mutual relations and roles in the EU. The regulators lay down the required safety information for allowing a substance on the market; industry provides this information to the regulators, which assess it. Industry gains this information through safety tests, which are sometimes developed and performed in-house, but often obtained from test developers or commissioned to contract research organizations (CROs). Industry then sends their substances to the CRO. Industry can also fund test developers, such as universities, to develop or validate certain desired tests. Test developers and CROs can also obtain funds from regulators, who can also indicate directly (i.e., not through industry) which test methods are needed. The limitations of current tests and possibilities of new technologies need to be communicated to the regulators, so these can adjust the safety information requirements accordingly. Such information can also be provided by industry, but will then not be free of commercial interests

In Europe, anyone may submit an application for validation of an alternative method to ECVAM. ECVAM then consults its network for the preliminary assessment of regulatory relevance (PARERE). PARERE then provides input, for example, as to whether the novel method measures a safety endpoint for which there is a regulatory need that is currently unsatisfied. To prevent that the regulators in PARERE discard a proposed method for further validation because, e.g., no potential use is foreseen, regular exchange between regulators and method developers on method needs and possibilities is desirable (Fig. 2). If regulatory relevance is identified, ECVAM validates the method. Approval is often quicker if the applicant submits supporting evidence. In addition, validation may be expedited when the applicants have taken good in vitro method practice (GIVIMP) guidelines into consideration, e.g., ensured solubility of tested chemicals at the tested concentrations. ECVAM reports positive validation outcomes to its scientific advisory committee, which then performs an independent scientific review. When this step is successful, a final recommendation report is submitted to the OECD, where a test guideline for the method is created. When embedded in an OECD test guideline, a novel model is then ready for regulatory use worldwide and thus ready for use in replacing animal testing. Regulatory agencies, such as ECHA and EFSA, can then easily include these methods in their guidelines (Fig. 2), as has been done very quickly after the acceptance of the Extended One Generation Reproduction Toxicity (EOGRT) test, for example. These guidelines (or guidance documents) describe which safety information they require from the industry to decide whether a chemical may be allowed on the market (Fig. 2), usually detailed to which methods are allowed. They are not legally binding, but are usually adhered to by the regulators and thus industry.

Validation of new in vitro models can also be challenging, as they cover only a small part of the body or functional system in vivo. This is also the case for the advanced in vitro systems discussed in this review, when applied to more complex safety endpoints such as repeated dose toxicity. A one-on-one comparison with the in vivo gold standard test to determine the predictive capacity is then not realistic. As a solution, integrated approaches to testing and assessment (IATAs) can be used, in which different alternative methods are combined to predict one endpoint. These IATAs currently form a challenge for the OECD, as multiple IATAs may be developed, consisting of different testing methods, which are not governed by a single test guideline. A new form of OECD guideline is, therefore, necessary to enable scientific acceptance of the advanced in vitro systems for more complex endpoints.

In summary, for worldwide regulatory acceptance of the currently emerging advanced in vitro methods:

- An exchange between regulators and developers is necessary, to exchange the regulatory needs (to avoid dismissal by PARERE) and technical possibilities.
- Test developers are strongly advised to take GIVIMP guidelines into consideration when preparing the validation package.
- The OECD has a challenge in finding a new way to formulate mutually accepted guidelines for IATAs consisting of these methods.

In the meantime, acceptance by regulators can be achieved on a case-by-case basis for special drugs, when well-justified.

## Models to exemplify progress and the current state-of-the-art

### Heart

Cardiotoxicity is a major cause of drug attrition and a substantial safety concern (Cross et al. 2015; Onakpoya et al. 2016). Certain aspects of cardiotoxicity such as long QT syndrome and arrhythmia can be accurately predicted by combining hERG channel inhibition data and QTc interval measurements in the heart’s electrical cycle (Wallis 2010). However, this leaves structural cardiotoxicity (i.e., direct damage to tissue) unaddressed. The underlying mechanisms of structural cardiotoxicity are poorly understood and current in vitro models cannot replicate it to an acceptable standard. Therefore, improved in vitro cardiotoxicity models are necessary.

A recurring theme between organ models is the lack of physiological relevance of 2D cultures using immortalised cell lines and dedifferentiated primary cells, which hold true for cardiotoxicity models. Furthermore, inter-species and inter-individual variation makes extrapolation of in vitro data to humans challenging. Stem-cell-derived cardiomyocytes (SC-CMs) are relatively novel models which help to overcome inter-individual and inter-species variation and are able to capture the phenotype of the donor cell, offering advantages over immortalised cell lines which represent only a single donor phenotype. SC-CMs have recently been shown to be accurate in predicting doxorubicin-induced cardiotoxicity severity and identifying the underlying pharmacogenetic mechanisms (Burridge et al. 2016; Mikaelian et al. 2010).

3D cardiac models show improved cell viability and enhanced structure and function (Edmondson et al. 2014; Nam et al. 2015). When developing 3D models, the origin and composition of the cells should be considered; in vivo, myocardial tissue comprises of 30% cardiomyocytes and 70% non-myocyte cells (NMCs, predominantly endothelial cells and fibroblasts). These NMCs are important in myocardial structure and function, as well as in development of drug-induced cardiovascular injury (Brutsaert 2003; Mikaelian et al. 2010; Souders et al. 2009). 3D models combining cardiomyocytes and NMCs were shown to be functionally superior to 2D models and could model calcium dyshomeostasis, mitochondrial disruption and loss of cell viability in response to cardiotoxicants (Pointon et al. 2013; Ravenscroft et al. 2016a, b). Microfluidic models of cardiotoxicity are under development, and they hold promise to improve physiological relevance by modelling vascularisation and structure which is not currently achievable in other models (Bhatia and Ingber 2014). Recently, the first 3D-printed heart-on-a-chip with an integrated sensing system for non-invasive electronic readouts was produced and successfully applied to study drug responses (Lind et al. 2016).

### Skin

The skin is a highly immunocompetent barrier and is important with regard to the absorption of drugs and chemicals and, therefore, dermal toxicity assessment. There are several approaches towards modelling the complexity of human skin. The most common model as a simple 2D monolayers of human keratinocytes that are routinely utilised for pre-clinical screening. 2D monolayer models do not recapitulate aspects of skin structure such as cornification and cannot model barrier function or immunological pathways. Complex models such as reconstructed human epidermis (RHE), a 3D organotypic model formed from primary cells are capable of forming a well-stratified epithelium which can model metabolism and barrier functions (Alépée et al. 2015). There are several commercially available RHE models which have been validated by EVCAM as an alternative to animal testing for assessing skin corrosion and skin irritation in a regulatory context while fulfilling the current OECD test guidelines (OECD 2015, 2014). Several studies have shown the RHE model to be superior to traditional 2D culture in terms of identification of allergic sensitisers (Gibbs et al. 2013) and modelling of immunological events in the epidermal layer (Ezendam et al. 2016). However, human skin is comprised of several layers, and this complexity is not addressed by RHE. Therefore, novel models which can recapitulate this complexity are required.

In line with other multi-cell models, full thickness models (FTMs) are composed of an epidermal and dermal layer. The epidermal layer is comparable to RHE and the dermal layer contains human dermal fibroblasts distributed throughout a collagen matrix. Studies have investigated the inclusion of melanocytes and MUTZ-Langerhans cells in an FTM showing improved modelling of sensitisation (Kosten et al. 2015) and also the inclusion of other cell types including endothelial cells (Tremblay et al. 2005) and hair follicles (Michel et al. 1999). In addition to multiple cell types, the extent of cell differentiation and the formation of other skin components such as the basement membrane is also important. Creating skin models with a continuous competent stratum corneum suitable for long-term culture is challenging, but would be useful for testing of subchronic and chronic toxicity. Lab-on-chip microsystems exhibit prolonged culture times as well as stable cell functions within a controlled microenvironment and a linkage with other organs (Maschmeyer et al. 2015). However, their predictivity and validation is yet to be assessed and should be the focus of further investigations.

### Liver

Hepatotoxicity is an important safety concern for industry. Xenobiotic metabolism enzymes are highly expressed in liver to deal with the first-pass dose of drugs or chemicals. This may produce chemically reactive metabolites in addition to the parent compound, both of which may cause toxicity, making hepatotoxicity complex to accurately model. Therefore, metabolic capacity is a crucial requirement for a valid model of hepatotoxicity and this is currently not fulfilled. This is exemplified by the fact that liver toxicity is among the leading causes of safety failures in clinical trials (Cook et al. 2014; Laverty et al. 2011; Onakpoya et al. 2016). A reliable in vitro hepatotoxicity model should resemble the in vivo phenotype as well as be suitable for long-term studies and high-throughput screening applications (Lauschke et al. 2016).

Human primary hepatocytes (hPH) are the gold standard of in vitro liver models, but are hindered in several respects: scarcity, since hPH may only be derived from surgical waste tissue or cadaveric liver; inter-individual variability, where the limited number of cells available from each donor and their finite lifespan can hinder repeat studies; and dedifferentiation, the loss of mature hepatic phenotype which occurs under standard cell culture conditions. Moreover, hPH cannot easily be expanded in vitro. Liver-derived cell lines such as HepG2 and HepaRG cells are available, but have limited metabolic capacity (Sison-Young et al. 2015). Recently, proteomic characterisation of hPHs, HepG2, HepaRG, and Upcytes revealed that HepaRG cells have the most similar protein profile to primary human hepatocytes (Sison-Young et al. 2015). However, in an assessment of the predictive capacity of hepatotoxicity, HepG2 was the most predictive, after the gold standard hPH (Sison-Young et al. 2016). These results indicate that none of the most commonly used hepatic models are satisfactory to fully model hepatotoxicity in vitro. Stem-cell-derived hepatocyte-like cells (SC-HLCs) are another emerging model, but currently show an immature hepatic phenotype. SC-HLCs retain the phenotype of the donor cell when reprogrammed presenting the opportunity to model rare phenotypes reproducibly, which may be more susceptible to DILI.

**Fig. 3:**
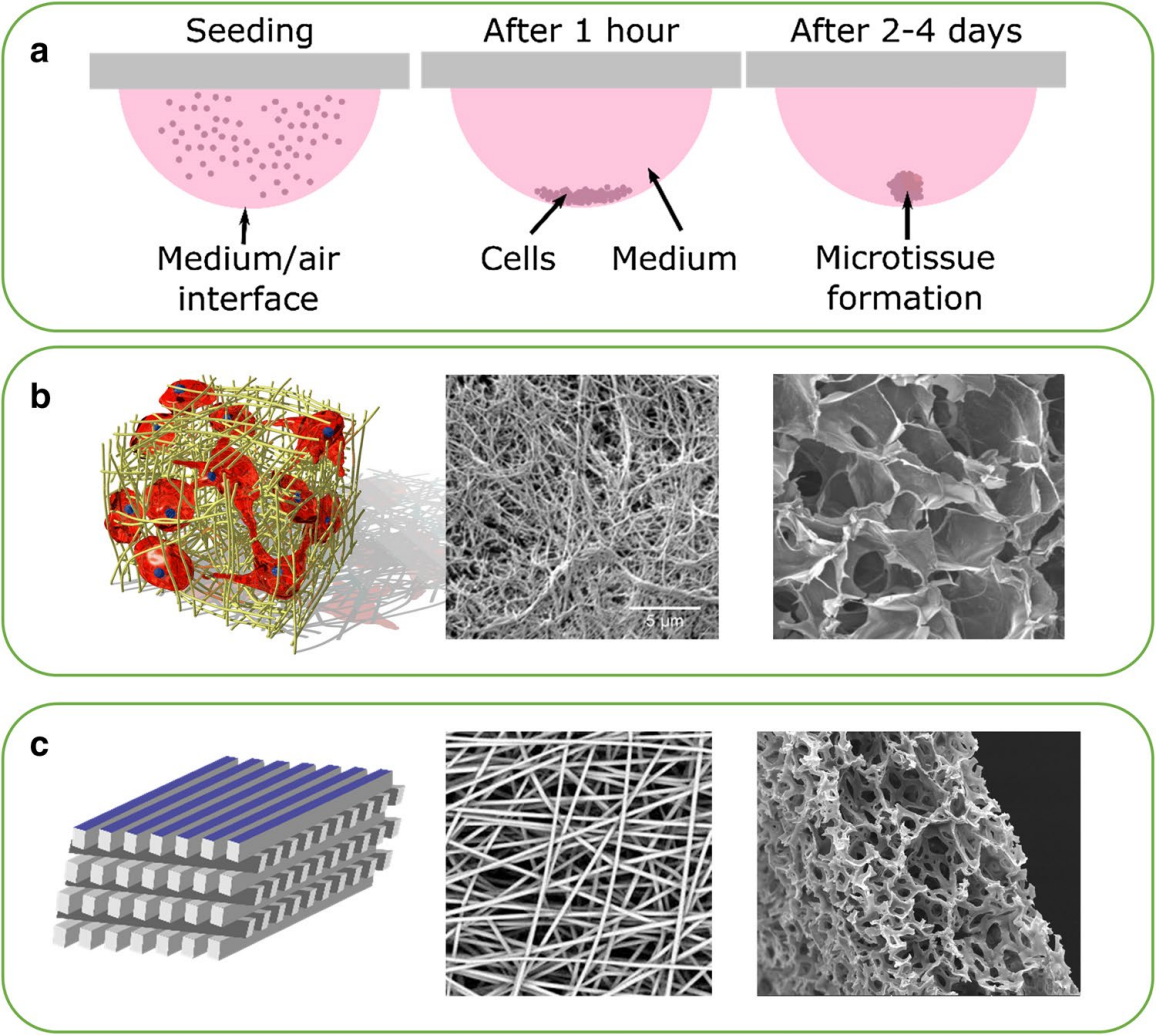
Approaches to enable 3D architecture for a cell culture production of spheroids in, e.g., hanging drops (**a**), use of hydrogels (**b**) of synthetic or natural materials, such as alginate (last picture in **B**), or use of scaffolds produced from natural materials or synthetically from polymers (**c**), which are either layered (first picture), electrospun (second picture) or polymerized in a sponge-like structure (third picture)

3D culture techniques such as spheroid culture (Fig. 3a) often show an enhanced phenotype with superior sensitivity and specificity in detecting DILI compounds over 2D systems (Bell et al. 2016; Berger et al. 2016; Kostadinova et al. 2013; Nguyen et al. 2016). However, similar caveats to the use of hPH in 2D models apply to 3D models and the use of an expandable cell line (e.g., SC-HLCs or immortalised cell lines) may be preferred in some cases. Spheroids form spontaneously under appropriate conditions and can be made from most extant hepatic models. It is likely that they will form a cornerstone of future hepatotoxicity testing.

In addition to 3D culture, the inclusion of non-parenchymal cells (NPCs) is necessary for modelling of DILI; inclusion of NPCs will allow modelling of complex liver toxicology such as fibrosis. NPCs can be incorporated into spheroid models (Bell et al. 2016). In addition, 3D bioprinting can reproduce the highly-organized liver architecture without use of a scaffold, with rapidly-forming tight junctions and extracellular matrix yielding solid microtissues that can model complex liver toxicity including fibrosis (Nguyen et al. 2016; Norona et al. 2016). Similar to the other organs described herein, microfluidic devices are under development and these may prove a fruitful avenue of research in the future.

## Mathematical contribution to the development of innovative toxicity models

Mathematical modelling can provide an alternative and complementary safety assessment platform for increasing mechanistic understanding, testing hypotheses in silico, predicting quantitative outcomes and contributing to the optimisation, design, and interpretation of innovative in vitro models. Indeed, such quantitative approaches have always informed and influenced pharmacology, formulating models at the interface of human physiology and the drug chemistry (Kenakin and Christopoulos 2011). The utility of quantitative modelling in drug classification and characterisation makes mathematics an important tool in drug discovery and is seen as an integral component to the advancement of toxicity testing (Krewski et al. 2007; Raies and Bajic 2016). The computational implementation of these quantitative, mathematical models, or in silico modelling, is now also used extensively as part of the pre-clinical drug development process (Visser et al. 2014) (Fig. 1). Mathematical models have been used to identify critical parameters involved in the metabolism of drugs such as acetaminophen using numerical, sensitivity, and timescale analysis as well identifying critical dose thresholds (Reddyhoff et al. 2015). This illustrates how modelling can be used to optimise in vitro design to ensure that relevant dosing regimens are studied and that the experimental model being used is physiologically relevant in terms of the expression of appropriate metabolising enzymes, for example. More directly, mathematical modelling has been used to optimise the design and operation of in vitro liver models such as hollow fibre membrane bioreactors by providing spatial information describing fluid and mass transport coupling in the cellular environment that would otherwise be experimentally expensive (Williams et al. 2013).

## Common themes between models

There common themes between advanced in vitro models, which may help facilitate their scientific acceptance and use. An important rule for these models is that they should be as complex as is required but as simple as possible. For example, a cell model should include the minimum number of cell types to recapitulate the in vivo physiology accurately. Adding more cell types can decrease reproducibility, increase costs and test failures, and lead to unnecessary interference with the test outcome. However, inclusion of multiple cell types, or features such as peristaltic movement of the tissue, can affect the tissue response to toxicants (Kim et al. 2012), presumably improving the accuracy of the prediction. The degree of accuracy required by a test, and how complex the system should be, depends on the question and on the phase within the risk assessment or drug development process. In early phases, decreased accuracy in favour of reduced costs are acceptable and simpler models may be suitable.

### Phenotyping

Determining which in vitro model to use for a question, particularly determining the appropriate complexity can be challenging; and proper phenotyping of advanced models can help make an informed decision. Such phenotyping of advanced models has been performed for advanced in vitro models of the liver, skin and heart (Berger et al. 2016; Fentem et al. 1998; Kostadinova et al. 2013; Mathur et al. 2015; Nguyen et al. 2016; Pointon et al. 2013; Reijnders et al. 2015; Wiegand et al. 2014).

The IMI-MIP-DILI consortium provides a recent example of detailed phenotyping. In this study, the proteomes of hPHs cultured in 2D and 3D spheroid culture were analysed. It was shown that 2D cultures developed a dramatically altered protein expression pattern within 16 h (Bell et al. 2016; Lauschke et al. 2016), while 3D spheroid cultures maintained an expression profile similar to hPH for 7 days (Bell et al. 2016). This finding indicates the greater physiological relevance of 3D cultures over 2D and suggests an improved ability to correctly identify toxicants early post-isolation of primary cells.

### Scaffolds in 3D culture

Another common theme is the use of scaffolds to support 3D tissue structures in advanced in vitro models (Fig. 3). Hydrogels are a type of scaffold comprised of water, extracellular matrix (ECM) proteins and growth factors that mimic the in vivo ECM. There are various hydrogel products available, both natural (e.g., collagen hydrogels, Matrigel, alginate) and synthetic (e.g., PuraMatrix). The natural hydrogels, notably the popular Matrigel, suffer from batch-to-batch variation. Synthetic hydrogels are well defined, have small lot-to-lot variability and can be adapted to direct functionality.

Scaffolds may also take the form of solid materials that provide a mechanical support for tissues, fabricated either from biological (e.g., collagen, fibrin, chitosan, agarose) or from synthetic (e.g., polystyrene, polycaprolactone, polyurethane) materials. The main advantage of the biological materials is their biocompatibility and flexibility, while the advantages of the synthetic materials are their consistency and controllability. Synthetic scaffolds can be fabricated with defined porosity, thickness, and rigidity for the intended tissue and test. Matrix stiffness can be sensed by the cells and this can affect the types of cell adhesions, cytoskeletal structure, cell proliferation, and other factors (Yamada and Cukierman 2007). Disadvantages common to all scaffolds include the challenge to visualise and analyse individual cells and the fact that they are not yet suitable for all cells.

Decellularised scaffolds are emerging biological scaffolds produced by removing cells from ex vivo tissues. Retention of the microvasculature in the ECM is an important advantage of decellularised scaffolds, allowing for easier emulation of vascular tissue. The addition of a vacuum to conventional decellularisation protocols based on enzymes or detergent, significantly reduces production time during decellularisation (Lange et al. 2015). Since availability of human tissue is limited, pig tissue is often used (Greco et al. 2015). The required tissue is taken from the pig and repopulated with human cells (e.g., ex vivo skin tissue to make 3D in vitro skin tissue). In recellularised tissue, cartilage is replaced by weaker collagen, which may lead to issues with structural integrity; furthermore, compatibility with human cells needs to be fully assessed, especially for the effects of molecular triggers that may differ in donor animals.

#### Scaffold-free 3D models

Spheroids are an example of a scaffold-free model that forms spontaneously when adherent cells are denied an attachment surface (Fig. 3). They are capable of producing an ECM when grown in hanging drops, ultra-low attachment conditions or in micropatterned plates (Bell et al. 2016; Kelm et al. 2003; March et al. 2015; Otsuka et al. 2004). Spheroids have an optimal size, maximally ~ 200 µm in diameter, to prevent necrosis in the centre due to diffusion limitations of nutrients and oxygen as these structures are not vascularised and are most often not subjected to flow. A scaffold-free system does not have the disadvantages of scaffolds, such as binding of test substances, batch-to-batch variability, and impedance of transport or diffusion from the scaffold. However, fluid flow and shear stress become difficult to incorporate in cultures lacking an anchored scaffold.

### Good cell culture practice

The general considerations of good cell culture practice (GCCP) (Coecke et al. 2007) and GIVIMP are pivotal to the advance of in vitro method systems. This includes genotyping of cells used in culture, ensuring sufficient solubility of test substances in the medium, and correcting for loss of test substance due to evaporation, protein binding, binding to plastic, etc. In many organ-on-a-chip models, polydimethylsiloxane (PDMS) is used as material for the microfluidic chip, even though hydrophobic chemicals are known to partition into this material quite extensively (Borysiak et al. 2013; Domansky et al. 2013). This material property not only leads to loss of test substance, but also to carry-over to the medium in a next test in the same chip.

### Other considerations

Finally, it should be stressed that the added value of a new advanced in vitro model should be shown, by comparing its outcomes to those of simpler extant models. If added value is shown, ideally, a comparison with a ‘gold standard’ should be made, deriving the sensitivity and specificity of the new advanced in vitro model. The 3D culture models discussed herein (heart, skin, and liver) have not yet been fully investigated in terms of their sensitivity and specificity at predicting adverse drug reactions. Such experiments typically use a panel of training compounds such as those used by the IMI-MIP-DILI consortium, where there is some understanding of the toxicological profiles of the compounds (Richert et al. 2016). By testing with a panel of training compounds at defined concentrations, novel models can be ranked in terms of sensitivity and specificity, where an ideal model would be both highly sensitive to adverse drug reactions while maintaining high specificity. Note that for drugs, this is easily achieved, as human data are available. For other chemicals, there may only be animal data, which may be less relevant. As stated before, it should be considered that comparing the results of one organ with those of a whole organism is not a fair comparison.

Developing a novel and ideal model requires effort, creativity, and innovation. Hence, collaboration between academia, regulators, and industry is necessary to develop such models for the mutual benefit of all. A collaborative approach to novel model development could lead to an open access database of protocols and methodologies for developing such models, which could also serve as reference for validation and standardisation of the created models (Alépée et al. 2014).

## Conclusions and recommendations

In conclusion, there is clearly a need for improved in vitro methods for toxicity testing for novel compounds such as those discussed herein. These first results with 3D models for skin, liver, and heart already demonstrate added value over traditional 2D toxicity testing methods. Nevertheless, it remains to be determined how complex the models and methods should be for each (regulatory or industry) question retaining simplicity where possible. More and better characterisation of the present models, in different variants, and comparisons with less or more complex models are necessary to fully understand, where they may best be deployed. This requires cross-talk between regulators, industry, and developers on what the questions are (“needs”), and what added value the different variants of models may have (“possibilities”). This calls for a large research effort and for meetings and workshops to exchange the needs with the possibilities.

Finally, new methods for characterisation of novel models should be standardised, comparison against a gold standard is not always possible or appropriate for novel models, and their proper handling can be challenging. Standardisation between groups for characterisation of comparable models should allow inter-model comparison and aid identification of true advances in the field.
